# Crash Severity Assessment: Is There a Need for Revisiting the Current Indicators?

**Published:** 2019-08

**Authors:** Homayoun Sadeghi Bazargani, Hadi Jalilvand, Aysan Mohammad Namdar, Yasin Sadeghi Bazargani, Mohammad Saadati

**Affiliations:** ^*a*^Road Traffic Injury Research Center, Tabriz University of Medical Sciences, Tabriz, Iran.; ^*b*^Faculty of Medicine, Tabriz University of Medical Sciences, Tabriz, Iran.

## Abstract

**Background:**

Many studies have been conducted to find the necessary components for assessing crash severity. Most of the prior studies relating to crash severity assessment mainly have been executed on the crash reports and road data. The aim of this study was to determine and rank the necessary indicators for developing a comprehensive and applicable indicator of crash severity based on expert reviews.

**Methods:**

95 participated in an online survey as a mixed method study to collect the ideas of experts in the field of crash severity during year 2019. The survey included 14 closed-ended and 18 open-ended questions whose closed-ended questions were based on Haddon’s matrix. Moreover, the questions were representative of potential risk indicators in relation to crash severity. The qualitative analyses were performed using STATA version 14 statistical software package.

**Results:**

Of 95 participants, 75 declared the country they had the most experience in, regarding traffic issues. The most number of responses belonged to Asia with 26 responses and the least number of responses was from Australia with 3 responses. The participants had interest/expertise in diverse fields; namely, the most stated field was road 34 (35.8%), followed by vehicle 16 (16.8%) and human public health 13 (13.7%). The least stated fields were Human mental health (1) and Ecosystem, environment and climate (0). With regard to academic degree and position of the participants, PhD (54.4%) and professor (28.3%) was the most prevalent. Among all the risk indicators in the survey, the majority (83.5%) totally agreed with including human damage in crash severity assessment, while 42 (38.9%) and 13 (11.9%) expressed this opinion on vehicle and environmental damage, respectively. Among the questions relating to human damage question, the majority totally agreed with including fatal injury (88.8%) and physical injury (84.1%), while there was less overall agreement on mental injury (34% totally agreed and 44.3% agreed). Some of the participants expressed concerns about including mental injury such as difficulty of measurement and less recognition in traffic investigation. Furthermore, Post-Traumatic Stress Disorder was referred to as a good but neglected indicator of human injury.

**Conclusions:**

In order to reach a comprehensive tool for measuring crash severity, the evaluation of human, vehicle, and environment could be essential. However, both the comprehensiveness as well as feasibility of measuring crash severity should be integrated.

**Keywords:**

Crash severity, Crash severity assessment, Traffic

